# Probiotics and yogurt modulate oxidative stress and fibrosis in livers of *Schistosoma mansoni*-infected mice

**DOI:** 10.1186/s12906-018-2406-3

**Published:** 2019-01-03

**Authors:** Manal F. El-Khadragy, Ebtesam M. Al-Olayan, Mohammed I. Y. Elmallah, Afra M. Alharbi, Hany M. Yehia, Ahmed E. Abdel Moneim

**Affiliations:** 1 Chair Vaccines Research of Infectious Diseases, Faculty of Science, King Saud University, Riyadh, Saudi Arabia; 2 Department of Zoology, Faculty of Science, King Saud University, Riyadh, Saudi Arabia; 3 Department of Zoology and Entomology, Faculty of Science, Helwan University, Cairo, Egypt; 4 Chemistry Department, Faculty of Science, Helwan University, Cairo, Egypt; 5 Department of Food Science and Nutrition, College of Food and Agriculture Sciences, King Saud University, Riyadh, Saudi Arabia; 6 Department of Food Science and Nutrition, Faculty of Home Economics, Helwan University, Cairo, Egypt

**Keywords:** Probiotics, Yogurt, *Schistosoma mansoni*, Fibrosis, Oxidative stress, MMP-9, Apoptosis

## Abstract

**Background:**

Considerable morbidity, mortality, and economic loss result from schistosomiasis infection. Deposition of *Schistosoma* eggs in the hepatic portal vein is considered as the main causative agent for the development of liver fibrosis and subsequent liver cirrhosis. Probiotics are exogenous and beneficial microorganisms to living hosts against the harmful effect of many parasites. Strong evidence suggests the importance of probiotics in the control strategy of helminth. The ultimate goal of this study is to evaluate the protective effect of probiotics and yogurt on *Schistosoma mansoni*-induced oxidative stress and hepatic fibrosis in mice.

**Methods:**

Mice were infected by tail immersion of schistosomal cercariae followed by an oral treatment with either probiotics or yogurt for one week before infection and immediately post-infection. Mice were scarified on day 56 following infection with *S. mansoni* and liver sample were obtained.

**Results:**

We showed that oral administration of probiotics or yogurt revealed a significant reduction in worm number, egg load, and granuloma size in liver tissue, which is mainly assigned to the decreased expression level of matrix metalloproteinases 9 (MMP-9) in liver tissue. A significant reduction in the oxidative stress markers-induced by *S. mansoni* infection including lipid peroxidation and nitrite/nitrate was also detected. The level of some antioxidant enzymes (superoxide dismutase, catalase, glutathione peroxidase and glutathione reductase) and reduced glutathione was greatly enhanced. Furthermore, treatment with probiotics or yogurt inhibited apoptosis in hepatic tissue, which is mainly assigned to the decreased expression level of caspases-3 in liver tissue.

**Conclusion:**

Our findings represent the promising anti-schistosomal activities of probiotics and yogurt.

**Electronic supplementary material:**

The online version of this article (10.1186/s12906-018-2406-3) contains supplementary material, which is available to authorized users.

## Background

Schistosomiasis or Billharzia, is a human helminth infectious disease caused by the parasite of the genus schistosoma. It affects more than 200 million people worldwide and causes more than 250,000 deaths per year [1]. Considering geographical distributions, there are different *Schistosoma* species that can infect human. *S. mansoni* and *S. hematobium* are mostly endemic to Africa and Middle East, which represents about 85% of the reported world cases [2]. The infectious phase (Cercariae) is developed particularly in fresh water areas, where the snails carrying the *Schistosoma* sporocytes exist. Cercariae penetrate the human skin and then migrate via blood circulation to the portal vein where the female parasite produces eggs. This leads to the appearance of acute and chronic symptoms such as fever, abdominal and anemia pain, exercise intolerance, and bloody diarrhea [3]. Worldwide, 10% of the intestinal schistosomiasis, *S. mansoni* infected people is associated with hepatic periportal fibrosis, hepatosplenomegaly, and esophageal varices as a result of long term exposure to the highly antigenic eggs [4]. Only *S. mansoni* and *S. hematobium* are found in Egypt [5]. It has been reported that eggs trapped in the host tissues are highly antigenic, which represents the major cause of pathology. The toxic components that continuously released by worm egg including soluble egg antigen (SEA) obviously harms host cells, promoting the formation of large inflammatory lesions called granulomas enclosing the eggs. These granulomas were suggested to be a protective mechanism for the neighboring cells against the devastating effect of the toxic egg components [6]. Additionally, SEA are implicated in the apoptosis induction in schistosomiasis infected tissue, however, the mechanism of apoptosis has not yet been fully understood [7]. Disturbance in the cellular antioxidant system, elevated level of oxidative stress such as lipid peroxidation-induced by the secretory egg components, and damage of hepatocyte membrane were also detected [8]. Furthermore, granulomas lead to fibrosis, which is characterized by the accumulation of the extracellular matrix (ECM) proteins like collagen, indicating the most serious symptoms of chronic schistosomiasis.

The current strategy in Egypt to control all forms of schistosomiasis mainly depends on chemotherapy by the only antischistosomal drug praziquantel (PZQ). This drug was introduced in clinical practice in the late 1970s. Several studies have been reported the chemistry, clinical efficacy, and side effects of PZQ [9]. However, the appearance of drug resistance strain of schistosoma was reported [10]. The resistance of *S. mansoni* strain toward PZQ treatment was revealed among villagers living in the Nile Delta region of Egypt [11]. This resistance has mostly been assigned to the long-term treatment with PZQ, indicating the increase of risk factors for the future development of drug resistance. A recent study also reported the appearance of PZQ-resistant schistosome isolates in China [12]. The inability of PZQ to destroy the immature parasites has been attributed to a large variety of unidentified host factors that prevent the maturation of the parasite and subsequent decrease of the drug efficacy [5]. Taking all together, the discovery as well as the development of new and effective preventative strategies for schistosomiasis is highly recommended.

Probiotics are referred to a wide range of symbiotic microorganisms isolated from gut, when administered conveys health improvement via modulation and restoration of gut microbiota [13]. Different types of bacteria are used as probiotics. The most commonly used bacteria include *Lactobacilli* and *Bifidobacteria* [14]. These bacteria have a symbiotic relationship with the human host. They colonize the mucus membrane present on epithelial cells of the gut where they inhibit the growth and attachment of harmful bacteria by producing bactericidal compounds (e.g. bacteriocins [proteinaceous toxins produced by bacteria to inhibit the growth of similar or closely related bacterial strain(s)], antibiotics, free fatty acids, hydrogen peroxide) against these harmful bacteria [15], regulate intestinal motility and mucus secretion [16], Additionally, probiotics have many metabolic importances such as regulate insulin homeostasis, fermentation of non-digestible food, biotransformation of certain xenobiotics and drugs and synthesis of some vitamins and short chain fatty acids. Probiotics may be a factor that can effectively suppress the growth and passage of several pathogens by enhancing the formation of intestinal mucosal barrier and mucosal immunity [17]. They are evidenced to be efficacious for the treatment of gastrointestinal disturbances, respiratory infections, and allergic symptoms. Furthermore, several studies have reported the potential effect of probiotics on parasites such as protozoans (e.g., *Cryptosporidium*, *Eimeria*) and worms (e.g., *Ascaris*, *Trichuris*) [16].

The mechanism by which probiotics work still remains unexplored. However, it has been reported that probiotics play a major role in the control of intestinal and non-gut parasite infections [16]. Adequate administration of probiotics in patients found to stimulate the immune response against pathogens [18]. Modulated the immune system by stimulating the host immune response to a variety of pathogens [19]. Probiotics also exhibit metal chelating activity. Therefore, probiotics are suggested to be a potential source of antioxidant compounds so that it can diminish the elevated level of reactive oxygen species (ROS), which is characteristic of gastrointestinal disorders.

The effect of probiotics on schistosomiasis was reported by Ghanem et al. [20]. In this study, the authors found that probiotics effectively restored liver function markers and displayed immunomodulatory effect. However, the impact of probiotics on pathological alternation, fibrosis and apoptosis those associated with schistosomiasis were not investigated yet. Hence in the present study, we investigated the protective effect of probiotics and yogurt on liver fibrosis and oxidative stress in mice infected with *S. mansoni*. Level of oxidative and antioxidative specific markers as well as the level of proapoptotic proteins in the hepatic homogenate of both untreated and treated (probiotics/yogurt) mice were also investigated. In the present study, we used *Lactobacillus acidophilus* ATCC 4356 and *Lactobacillus delbrueckii* subsp. *bulgaricus* DSM 20080 as they are already available in many commercial products.

## Methods

### Experimental animals

Forty-nine male CD-1 Swiss albino mice of an initial body weight of 20 ± 5 g were obtained from the Schistosome Biology Supply Center (SBSC) of the Theodor Bilharz Research Institute (TBRI), Giza, Egypt. Mice were maintained on a standard commercial pellet diet and housed under standard caging conditions at 22–25 ± 1 °C. Animals were bred under specified pathogen-free conditions and permitted ad libitum consumption of water. The animal experiments were conducted at the TBRI animal unit in accordance with international ethical guidelines after approval by the institutional ethical committee of TBRI and the study protocol and animal handling procedure are approved by the Institutional Animal Ethics Committee at Helwan University (approval No. HU2016/Z/01).

### Starter cultures

*Lactobacillus acidophilus* ATCC 4356 and *Lactobacillus delbrueckii* subsp. *bulgaricus* DSM 20080 were provided by ATCC (Manassas, VA, USA). To obtain viable and active bacterial strains, both bacterial strains were separately inoculated in de Man, Rogosa, Sharpe broth media (MRS) (Oxoid CM0359; Thermo Fisher Scientific, UK) and incubated at 30 °C for 24 h. The cultures were centrifuged at 4000 rpm for 10 min and the cell pellets were washed twice in phosphate buffer saline (PBS). Finally, cells were resuspended in PBS and mixed at a ratio of 10^8^:10^8^ cfu/g before feeding to mice through oral gavage tubes.

### Yogurt production

Milk was obtained from a retail farm located in Qalyubia Government, Egypt. Milk was pasteurized at 85 °C for 10 min, cooled to 4 °C, and then warmed in a water bath up to 42 °C. The starter cultures (*L. acidophilus* ATCC 4356 and *L. delbrueckii* subsp. *bulgaricus* DSM 20080) were inoculated at the ratio of 10^8^:10^8^ cfu/g into the cooled milk, incubated at 37 °C for 5 h, and finally kept at 4 °C [21].

### Infection of mice

*Schistosoma mansoni* cercariae were obtained from SBSC at TBRI. Each mice received approx. 70–75 cercariae using the tail immersion procedure described by Oliver and Stirewalt [22].

### Experimental design

Mice were divided into seven groups (*n* = 7). Group I served as the normal (non-infected) control. Groups II-VII were infected with *S. mansoni*. On day 46 post-infection (PI), PZQ group (Group III) was orally treated in the chronic phase only with a single dose of PZQ (Praziquantel-Sedico Pharmaceutical Co. 6th of October City, Egypt) at 500 mg/kg body suspended in olive oil for two successive days. Groups IV and VI (pretreatment groups) were administered 100 μL of probiotics solution or yogurt, respectively, by oral gavage. Groups V and VII (treatment groups) were administered 100 μL of probiotics solution or yogurt, respectively, by oral gavage. Oral administration of either probiotics or yogurt started one week preinfection in Groups IV and VI or on the first day PI in Groups V and VII. The doses of probiotics and yogurt were calculated according to those used in a preliminary study conducted to examine the antioxidant properties of the compounds while the PZQ dose was calculated as previously described by Dkhil et al. [23]. Group II served as the infected (untreated) control. Table 1 summarizes the animal groups.

**Table 1:** Experimental design: in vivo

Group	Name	Treatment	Dose	Duration of treatment
I	Normal group	–	–	–
II	Infected-untreated group	–	–	–
III	PZQ-treated group	PZQ	500 mg/kg	2 days
IV	Pre-probiotics treated group	Probiotics	100 μL/mouse	63 days
V	Probiotics-treated group	Probiotics	100 μL/mouse	56 days
VI	Pre-yogurt treated group	Yogurt	100 μL/mouse	63 days
VII	Yogurt-treated group	Yogurt	100 μL/mouse	56 days

Mice were decapitated on day 56 PI under mild ether anesthesia followed by collection of blood immediately for serum analysis. Livers were dissected and washed twice in ice-cold 50 mM Tris–HCl, pH 7.4. To obtain a 10% (*w*/*v*) liver homogenate, livers were homogenized in ice-cold 50 mM Tris–HCl, pH 7.4 then centrifuged at 900×*g* for 10 min at 4 °C. Supernatants were collected and used for subsequent biochemical analysis. Total protein content in liver homogenate was determined according to the method of Lowry et al. [24].

### Liver perfusion and ova count

Mature worms from hepatic portal vein and liver were recovered by perfusion technique described by Aly and Mantawy [25]. Following perfusion, the recovered worms were left to sediment for 20 min in a small Petri dish to facilitate sex identification, examination, and counting. The degree of protection or the percent reduction in parasite load was calculated according to the following formula:

$$ \mathrm{P}=\mathrm{C}-\mathrm{T}/\mathrm{CX}\;100,\mathrm{where} $$

P = is the degree of protection.

C = is the mean number of worms recovered from untreated infected mice.

T = is the mean number of the worms recovered from treated mice.

Egg burden was determined by overnight incubation of liver tissue in 5% KOH at 37 °C and then the recovered eggs were counted in 50 μl aliquots. The experiment was measured in triplicate.

### Liver function test

The level of liver transaminases, alanine aminotransferase (ALT) and aspartate aminotransferase (AST) was determined in blood serum according to the method of Reitman and Frankel [26]. Enzyme activities were assessed by estimating the amount of pyruvate or oxaloacetate formed in the derivative form of 2,4-dinitrophenylhydrazine. The activity of alkaline phosphatase (ALP) in serum was determined by the method of Belfield and Goldberg, using kits provided by Randox Laboratories Co. [27]. The method of Schmidt and Eisenburg was employed to assay the total serum bilirubin [28].

### Biochemical analysis of oxidative stress markers

Lipid peroxidation (LPO) in hepatic tissue was estimated by incubating 1 mL of thiobarbituric acid (0.67%; Sigma-Aldrich, USA) and 1 mL of 10% trichloroacetic acid (TCA; Sigma-Aldrich, USA) in a boiling water bath for 30 min. The amount of detected malondialdehyde (MDA) at 535 nm is directly related to the levels of thiobarbituric acid-reactive substances. [29]. The level of nitrite/nitrate in liver homogenate was assayed by Griess reagent in an acidic medium. The resulting diazotized sulfanilamide (Alfa Aesar; Germany) couples with N-(1–naphthyl) ethylenediamine (Alfa Aesar; Germany) to form a compound with a bright reddish purple color that can be read at 540 nm [30]. In addition, the level of hepatic reduced glutathione (GSH) was determined by the reduction of Ellman’s reagent [5,5′-dithiobis-(2-nitrobenzoic acid)] with GSH to form a yellow compound that can be measured at 405 nm. [31].

### Enzymatic antioxidant status

The enzyme activity of superoxide dismutase (SOD) in liver homogenates was assessed by measuring the suppressive capability of SOD to phenazine methosulfate-mediated by the reduction of nitroblue tetrazolium (NBT; Alfa Aesar; Germany) dye. Catalase (CAT) activity in hepatic homogenates was determined by mixing 50 μL of homogenates with 30 mM H_2_O_2_ in 50 mM potassium phosphate buffer (pH 7.8). The consumption of H_2_O_2_ was measured at 340 nm for 120 s at 30 s intervals. Glutathione reductase (GR) activity was indirectly tested by measuring the amount of reduced glutathione formed in the reaction mixture in the presence of NADPH (Sigma-Aldrich, USA) at 340 nm. NADPH reduction can be measured by the decrease in absorbance. Hepatic glutathione peroxidase (GPx) activity was measured using the method described by Paglia and Valentine [32]. Briefly, GPx reduces H_2_O_2_ via conversion of reduced glutathione (GSH) into its oxidized form (GSSG). GPx activity can be monitored by the reduction of GSSG back into GSH using glutathione reductase and NADPH. The oxidation of NADPH to NADP^+^ is accompanied by a decrease in absorbance at 340 nm.

### Histopathological examination

The collected liver sections were fixed in 10% Neutral-buffered formalin and embedded paraffin blocks. 5 μm sections of the fixed samples were prepared and stained with hematoxylin and eosin as described by Fischer et al., [33]. Granuloma size was measured in *S. mansoni*-infected rats. The size (μm^2^) was determined from the average of length and width. At least, one hundred granulomas were examined in each group using ImageJ software (U.S. National Institutes of Health, Bethesda, MD, USA).

### Immunohistochemical analysis of MMP-9 and caspase-3

Matrix metallopeptidase 9 (MMP-9) and caspase-3 were analysed by immunolocalization technique on 3- to 4- μm thick of liver sections. A primary antibody was omitted for negative controls. Briefly, liver sections were incubated for 1 h with mouse anti-MMP-9 or anti-caspase-3 (Santa Cruz Biotechnology, Santa Cruz, CA, USA) at dilution of 1:150) in Tris buffered saline (TBS)/bovine serum albumin (BSA) 1%. Anti-mouse biotinylated secondary antibody (Biotinylated Link Universal–DakoCytomation kit, supplied ready to use) was added and incubated for 20 min. Subsequently, horseradish peroxidase conjugated with streptavidin (DakoCytomation kit, supplied ready to use) was added for an additional 20 min. A reddish to brown color was developed following addition of 3-amino-9-ethylcarbasole (AEC) (DakoCytomation kit, supplied ready to use) for 20 min. Specimens were subjected to hematoxylin counterstaining for 60 s and mounted using Aquatex fluid (Merck KGaA, Germany). To exclude procedural artifacts among different experimental animal groups, all liver sections were treated with the same amount of antibodies under the same conditions.

Then, the cells were counted manually on the obtained images by two blinded technicians independently, which were then processed in ImageJ software (U.S. National Institutes of Health, Bethesda, MD, USA). After counting, the examined images were cropped, scaled to μm and separated by color channel, and artifacts were removed. The numbers of cell were expressed as counts/mm^2^. The ImageJ cell counter tool recorded mouse clicks on cells that were labeled with colored dots. Furthermore, the color intensity was graded as very weak, weak, medium, or strong.

### Determination of apoptotic markers in liver tissue

The level of apoptotic protein markers, Bax and Bcl-2 in liver homogenates were prepared in lysis buffer and analyzed using ELISA kits (R&D Systems Inc.) according to the manufacturer’s instructions. The levels of Bax and Bcl-2 were expressed as ng/mg protein.

### Molecular analysis (real-time qPCR)

Extraction and purification of total RNA from the liver tissues was performed using an RNeasy Plus Minikit (Qiagen, Valencia, CA). Random primers were designed for cDNA synthesis using the Script cDNA synthesis kit (Bio-Rad, CA). All primers (GPx1, SOD2, Bcl-2, and Bax) were obtained from Jena Bioscience GmbH (Jena, Germany) and their specificities were ensured by NCBI Blast tool. The primer sequences are listed in Table 2 as previously listed by Almeer et al. [34]. Gene expression analysis was performed in an Applied Biosystems 7500 Real Time PCR Instrument using Power SYBR Green (Life Technologies, CA). cDNA samples were prepared in triplicate. The typical thermal profile for the PCR reaction conditions were initiated by denaturation step at 95 °C for 4 min, followed by 40 cycles of amplification at 94 °C for 60 s (denaturation) and 55 °C for 60 s (annealing and extension). β-actin gene was used as a reference. ΔCt was calculated by subtracting the β-actin Ct from each test Ct.

**Table 2:** Primer sequences of genes analyzed in real time PCR

Name	Accession number	Sense (5′---3′)	Antisense (5′---3′)
β-actin	NM_031144.3	GGCATCCTGACCCTGAAGTA	GGGGTGTTGAAGGTCTCAAA
SOD2	NM_001270850.1	AGCTGCACCACAGCAAGCAC	TCCACCACCCTTAGGGCTCA
CAT	NM_012520.2	TCCGGGATCTTTTTAACGCCATTG	TCGAGCACGGTAGGGACAGTTCAC
GPx1	NM_017006.2	CGGTTTCCCGTGCAATCAGT	ACACCGGGGACCAAATGATG
Bcl-2	NM_016993.1	CTGGTGGACAACATCGCTCTG	GGTCTGCTGACCTCACTTGTG
Bax	NM_017059.2	GGCGAATTGGCGATGAACTG	ATGGTTCTGATCAGCTCGGG

The abbreviations of the genes; *SOD2* Manganese-dependent superoxide dismutase (MnSOD), *CAT* Catalase, *GPx* Glutathione peroxidase, *Bcl-2* B-cell lymphoma 2, *Bax* Bcl-2-like protein 4

### Statistical analysis

Data were expressed as mean ± standard error of the mean (SEM). All numerical data were analyzed using One-way ANOVA. Duncan’s test using a statistical package (SPSS version 20.0) was used to perform the statistical comparisons between groups. *p* < 0.05 was considered significant for all statistical analysis in the present study.

## Results

**Fig. 1:**
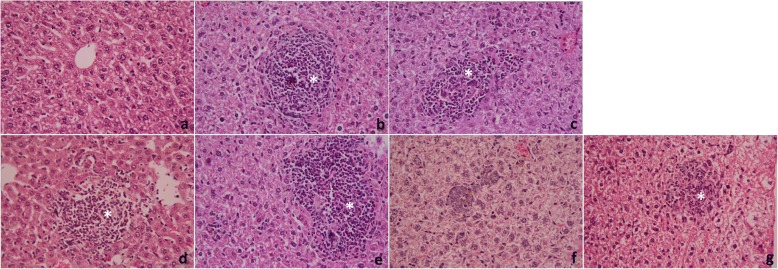
Sections of mouse livers 56 days post-infection with Schistosoma stained with hematoxylin and eosin. **a**: Control group showing normal liver architecture with normal hepatocytes and central vein. **b**: Untreated infected group, showing inflammatory cells, granulomatous lesions (asterisk), and focal areas of necrosis with condensed of connective fibrous tissue. **c**: PZQ (500 mg/kg body weight) + infection group, showing granulomatous lesions (asterisk) with decreased diameter and condensed connective fibrous tissue. **d**: Pre-Probiotics+infection group, showing inflammatory cells localized around the granuloma (asterisk) with less fibrous tissue. **e**: Probiotics+infection group showing a massive number of leucocytes surrounding area of granuloma (asterisk) with less fibrous tissue. **f**: Pre-Yogurt+infection group, showing inflammatory cells localized around the granuloma with less fibrous tissue. **g**: Yogurt+infection group showing massive number of leucocytes surrounding the area of granuloma (asterisk) with less fibrous tissue (400x)

Our results revealed that oral administration of yogurt or probiotic significantly (p < 0.05) reduced the number of recovered worms from *S. mansoni*-infected animal groups. However, the time-dependent efficacy of yogurt was more significant than probiotics. The worm load and ova count in untreated infected and treated infected mice are presented in Table 3. In infected treated mice, the count number of ova/g liver was also decreased in a similar manner. This results in a marked decrease in granuloma size and count, particularly in yogurt-treated mice. Histopathological investigation detected the formation of both cellular and fibrous granuloma with central apoptotic cells confirming the parasitological findings. Moreover, the appearance of condensed connective fibrous tissue, and a centrally localized embedded egg with leukocyte infiltration at the periphery in the hepatic parenchyma cells were also observed. In treated groups, apparent ameliorations were noticed (Fig. 1). This was represented in the reduction in epithelioid cells formation, fibrous tissues, and number of centrally localized eggs.

**Table 3:** Effects of probiotics or yogurt administration on worm burden, liver tissue eggs load, granuloma size and count of *S. mansoni* infected mice

Groups	Mean number of worms ± SEM	Reduction on worm burden (%)	Ova count in liver (ova/mg tissue) ± SEM	Reduction on ova count (%)	Granuloma size (μm^2^)	Granuloma number per area (mm)
Vehicle control	17.4 ± 2.47	–	11.4 ± 1.08	–	1,103,658 ± 2106	11.6 ± 2.3
PZQ	3.8 ± 1.12^a^	78.2	2.3 ± 0.71^a^	79.8	864,627 ± 1137^a^	6.2 ± 1.7^a^
Pre-Probiotics+infection	5.6 ± 1.41^a^	67.8	3.1 ± 0.21^a^	72.8	984,273 ± 1637^a^	8.1 ± 1.2^a^
Probiotics+infection	7.0 ± 0.89^a^	59.8	5.2 ± 0.39^a^	54.4	1,067,435 ± 2318	9.4 ± 2.3^a^
Pre-Yogurt+infection	4.8 ± 1.28^a^	72.4	2.7 ± 0.34^a^	76.3	361,474 ± 864^a^	5.8 ± 2.1^a^
Yogurt+infection	6.2 ± 1.06^a^	64.4	4.3 ± 0.28^a^	62.3	635,417 ± 1268^a^	6.9 ± 1.8^a^

Values are means ± SEM (*n* = 7). ^a^*p* < 0.05, significant change with respect to Vehicle control

**Fig. 2:**
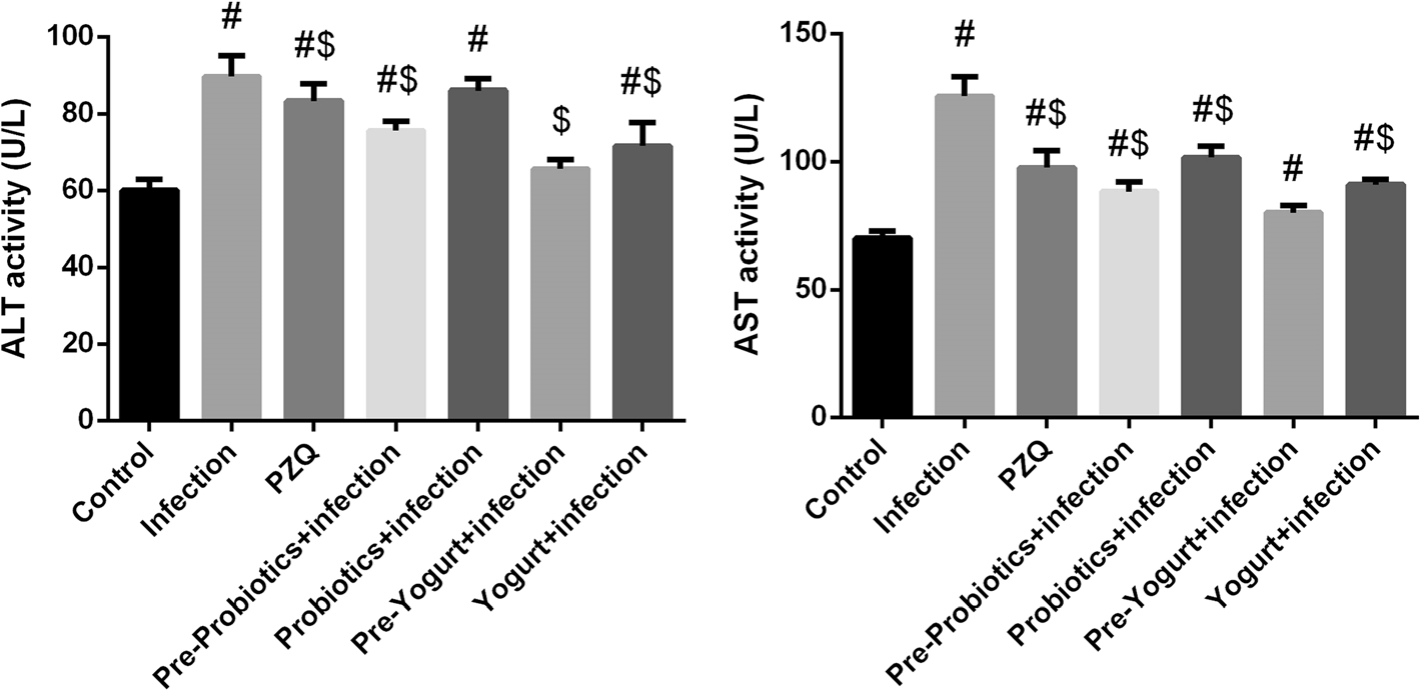
Effect of probiotics, yogurt, and PZQ on serum transaminase activities of control and experimental groups. Values are mean ± SEM (*n* = 7). ^#^*p* < 0.05, significant change with respect to Control group; ^$^*p* < 0.05, significant change with respect to Vehicle control group. *ALT* alanine transaminase and *AST* aspartate transaminase

The elevated level of liver function enzymes in *S. mansoni*-infected mice is quit supports the histopathological findings (Fig. 2). PZQ treatment group did not reverse the elevated transaminase levels. In contrast, the level of transaminase in yogurt- and probiotics-treated mice significantly improved compared to the non-treated infected group, whereas pretreatment with yogurt restored ALT level, when compared to control group. Notable, probiotics and yogurt administered to normal mice did not cause significant change in liver function markers (data not shown).

**Fig. 3:**
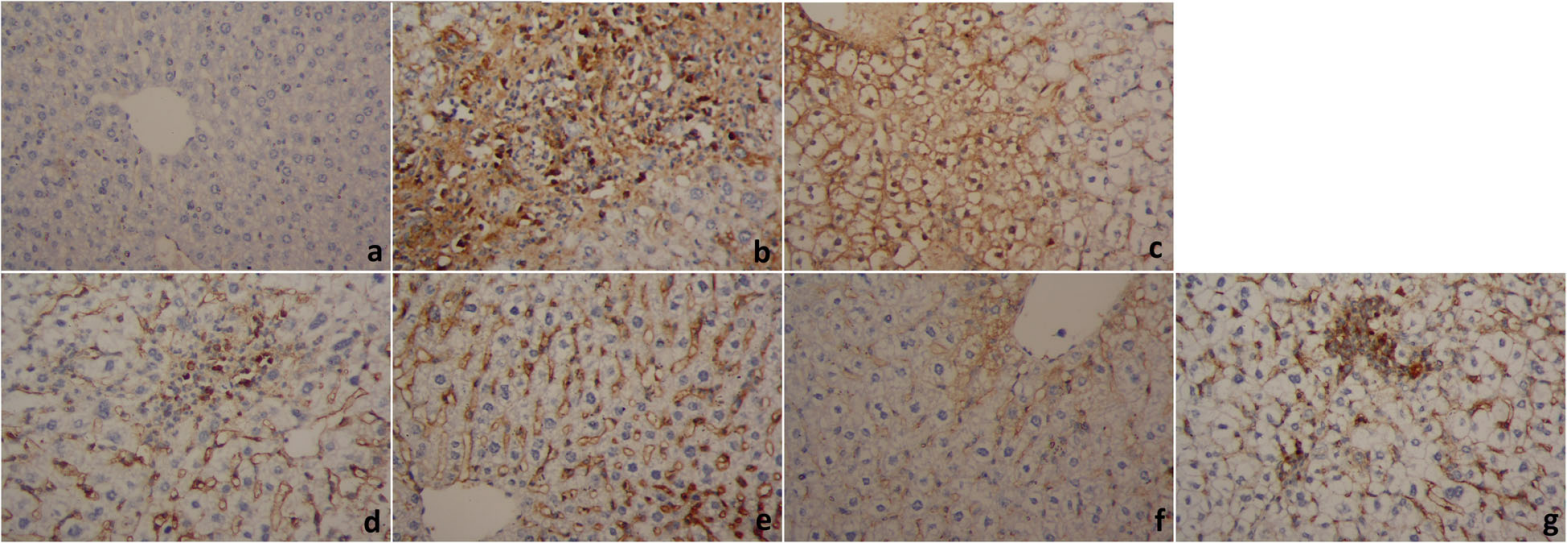
Livers of Schistosoma-infected mice stained with MMP-9 antibody, **a**: Control group, showing negative (−ve) immunoreactivity to MMP-9 in the hepatocytes and central vein. **b**: Vehicle control group, showing strong positive (+ve) immunoreactivity to MMP-9 in the hepatocytes. **c**: PZQ (500 mg/kg body weight) + infection group, mild immunoreactivity to MMP-9 in the hepatic tissues adjacent to granuloma. **d**: Pre-Probiotics+infection group, showing positive (+ve) immunoreactivity to MMP-9 in the hepatic tissues surrounding the granuloma. **e**: Probiotics+infection group, positive (+ve) immunoreactivity to MMP-9 in the hepatocytes adjacent to the fibrous granuloma in the hepatic tissues peripheral to the granuloma. **f**: Pre-Yogurt+infection group, showing positive (+ve) immunoreactivity to MMP-9 in the hepatic tissues surrounding the granuloma. **g**: Yogurt+infection group, positive (+ve) immunoreactivity to MMP-9 in the hepatocytes adjacent to the fibrous granuloma in the hepatic tissues periphery to the granuloma (400x)

Immunohistochemical analysis detected no significant immunoreactivity to MMP-9 in the hepatic tissues of the control (Fig. 3). The granulomated liver tissue of the untreated infected group displayed a marked immunoreactivity to MMP-9 as well as of PZQ treated animals (Additional file 1). Conversely, an obvious reduction of the immunostaining intensity to MMP-9 in the hepatic tissue-associated granulomas of yogurt- and probiotics-treated groups was detected. These results indicated that both yogurt and probiotics exhibit an antifibrotic effect, which is more significant considering yogurt treatment rather than treatments with probiotics. However, this effect did not exhibit in normal liver.

**Fig. 4:**
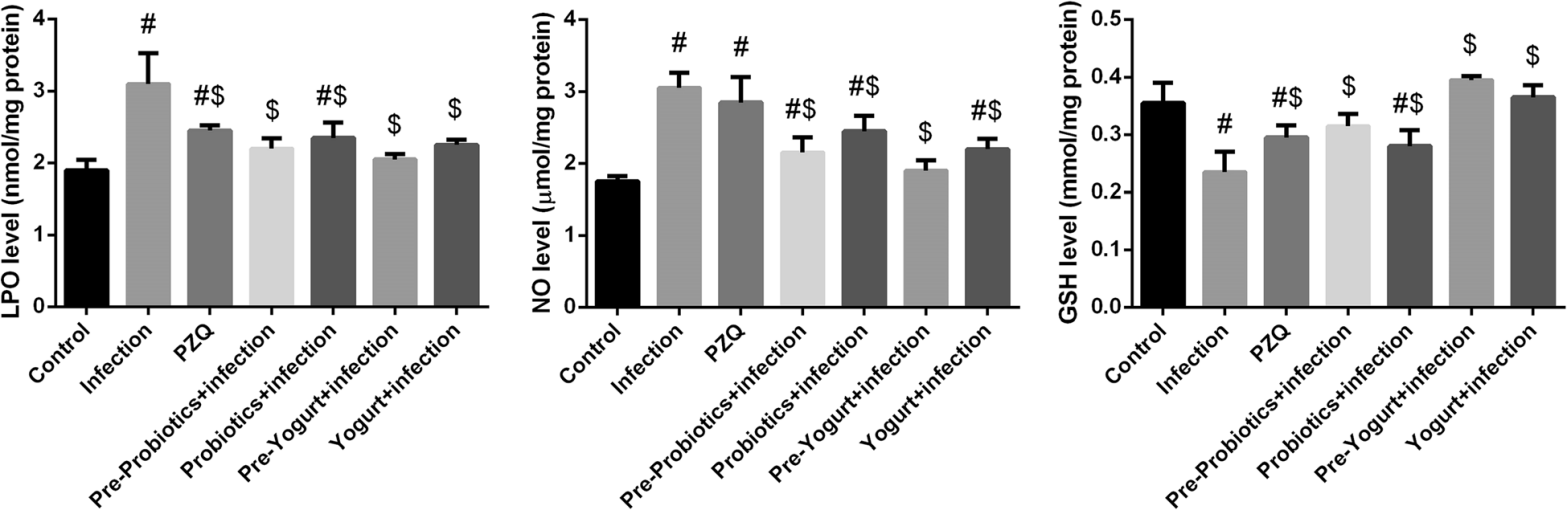
Effect of probiotics, yogurt, and PZQ on oxidative stress markers of control and experimental groups upon Schistosoma infection. Values are mean ± SEM (*n* = 7). ^#^*p* < 0.05, significant change with respect to Control group; ^$^*p* < 0.05, significant change with respect to Vehicle control group. *LPO* lipid peroxidation, *NO* nitric oxide and *GSH* glutathione

**Fig. 5:**
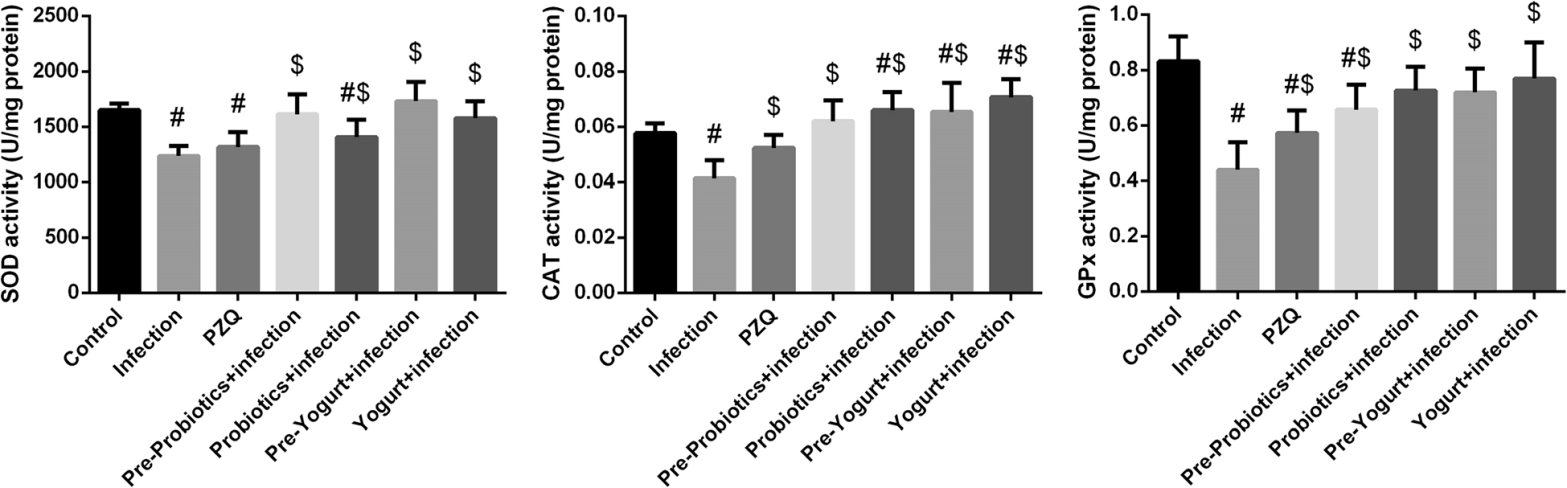
Effect of probiotics, yogurt, and PZQ on hepatic antioxidant enzyme activities of control and experimental groups upon Schistosoma infection. Values are mean ± SEM (*n* = 7). ^#^*p* < 0.05, significant change with respect to Control group; ^$^*p* < 0.05, significant change with respect to Vehicle control group. *SOD* superoxide dismutase, *CAT* catalase and *GPx* glutathione peroxidase

The level of oxidative stress markers including MDA, NO, and GSH in liver tissues was determined to investigate the effect *S. mansoni*-induced oxidative stress in mice (Fig. 4). It was shown that the level of MDA and NO was significantly (*p*<0.05) increased accompanied with a marked reduction of GSH compared to control mice. On the other hand, the reduction level of GSH and the formation of both MDA and NO were obviously decreased in yogurt and probiotics treated groups. To confirm the role of schistosoma infection in the perturbation of the antioxidant system in hepatic tissue, the activity of the antioxidant enzymes SOD, CAT, GST, GPx, and GR was determined (Fig. 5). *S. mansoni*-infected mice revealed significant (*p* < 0.05) reduction in the antioxidant enzyme activities when compared to control group. However, administration of yogurt or probiotics markedly (*p* < 0.05) enhanced the activity of the aforementioned antioxidant enzymes compared to the activities in the untreated infected mice. Noticeable, probiotics and yogurt administered to normal mice decreased MDA and increased GSH and antioxidant enzyme activities. But, the differences did not change significantly (data not shown).

**Fig. 6:**
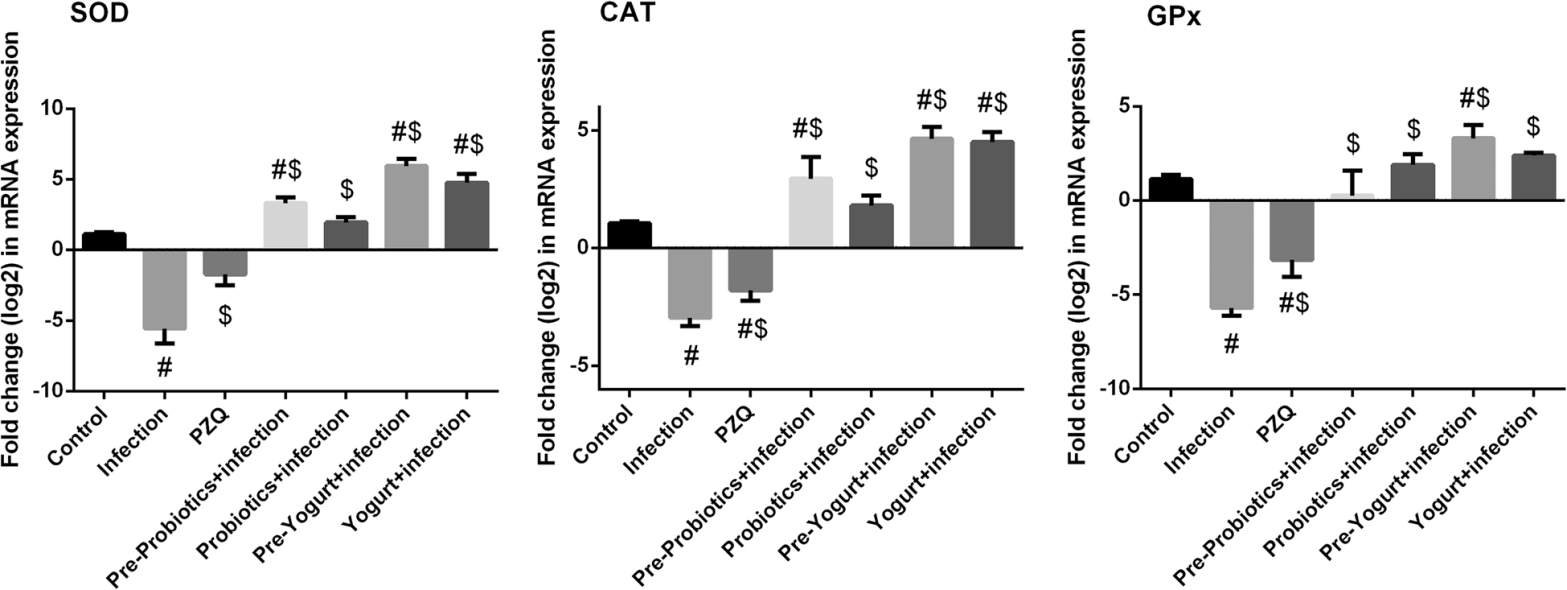
Effect of probiotics, yogurt, and PZQ on hepatic mRNA expression of the corresponding genes in control and experimental groups upon Schistosoma infection. Results (mean ± SEM of three assays) were normalized to β-actin RNA level and are shown as fold induction (in log2 scale) relative to the mRNA level in the control. SOD2: superoxide dismutase CAT: catalase and GPx1: glutathione peroxidase 1

Analyzing the gene expression profile of SOD2, CAT, and GPx1 in the hepatic tissue of *S. mansoni*-infected mice by quantitative real-time PCR indicated a significant downregulation of the tested enzymes, which is quit consistent with the biochemical results. Interestingly, animal groups treated with yogurt or probiotics exhibited a significant upregulation of the investigated enzymes (Fig. 6).

**Fig. 7:**
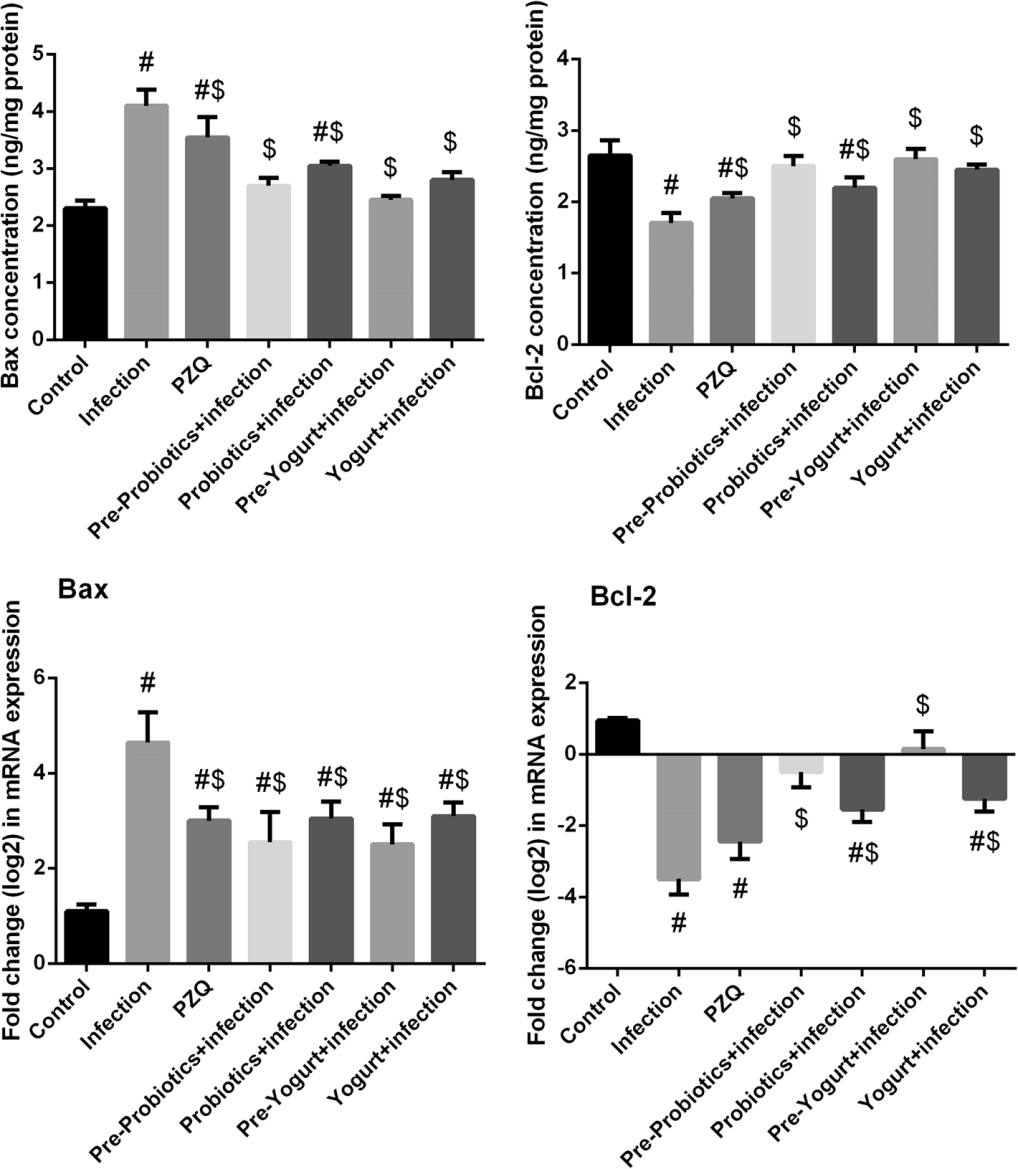
Effect of probiotics, yogurt, and PZQ on hepatic expression of Bax and Bcl-2 in control and experimental groups, **a**: Protein expression determined by ELISA method. **b**: mRNA expression determined by RT-PCR method. Results (mean ± SEM of three assays) of RT-PCR were normalized to β-actin RNA level and are shown as fold induction (in log2 scale) relative to the mRNA level in the control. ^#^*p* < 0.05, significant change with respect to Control group; ^$^*p* < 0.05, significant change with respect to Vehicle control group. Bax: bcl-2-like protein 4 and Bcl-2: B-cell lymphoma 2

The hepatoprotective effect of both yogurt and probiotics administration via investigation of their antiapoptotic properties was tested, that are represented in the determination of the expression level of Bcl-2 and Bax proteins in liver. Regarding the untreated *S. mansoni*-infected mice, the expression level of Bcl-2 and Bax (Fig. 7a) showed a marked (*p* < 0.05) upregulation. In contrast, administration of yogurt or probiotics prior- and post-infection with *S. mansoni* possessed a significant increase in the expression level of the pro-apoptotic Bcl-2 and a significant decrease in the expression level of Bax. These results were confirmed by analyzing the gene expression profile of both proteins (Fig. 7b). Noticeable, probiotics and yogurt administered to normal mice did not change the protein levels of Bcl-2 and Bax. However, at the molecular level, Bcl-2 expression was significantly increased (data not shown).

**Fig. 8:**
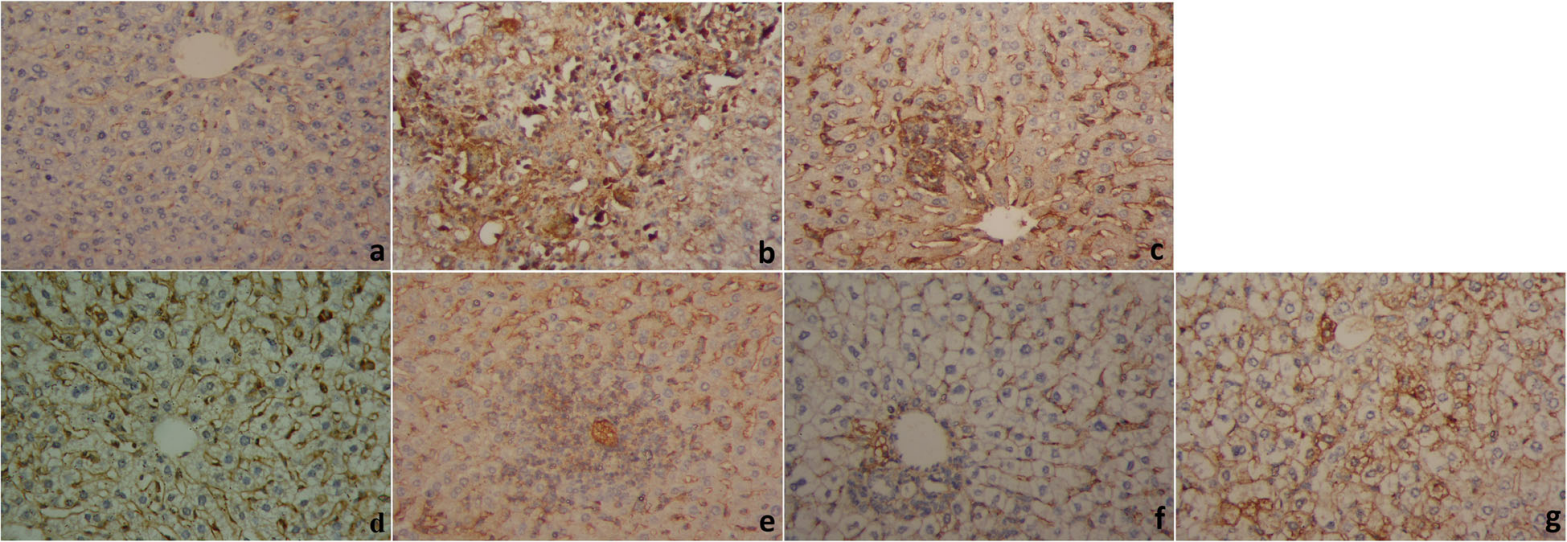
Livers of Schistosoma-infected mice stained with caspase-3 antibody, **a**: Control group, showing negative (−ve) immunoreactivity to caspase-3 in the hepatocytes and central vein. **b**: Vehicle control group, showing strong positive (+ve) immunoreactivity to caspase-3 in the hepatocytes. **c**: PZQ (500 mg/kg body weight) + infection group, moderate immunoreactivity to caspase-3 in the hepatic tissues adjacent to granuloma. **d**: Pre-Probiotics+infection group, showing mild positive (+ve) immunoreactivity to caspase-3 in the hepatic tissues surrounding the granuloma. **e**: Probiotics+infection group, moderate positive (+ve) immunoreactivity to caspase-3 in the hepatocytes adjacent to the fibrous granuloma in the hepatic tissues peripheral to the granuloma. **f**: Pre-Yogurt+infection group, showing mild positive (+ve) immunoreactivity to caspase-3 in the hepatic tissues surrounding the granuloma. **g**: Yogurt+infection group, moderate positive (+ve) immunoreactivity to caspase-3 in the hepatic tissues peripheral to the granuloma (400x)

Immunohistochemical analysis displayed an obvious increase in the immunostaining intensity signal for of the apoptotic protease caspase-3 in hepatic tissue of infected mice (Fig. 8). This intensity was reduced, indicating a significant decrease in the number of caspase-3 positive hepatocytes in animal groups treated with yogurt or probiotics prior- and post-infection (Additional file 1).

## Discussion

*S. mansoni* is considered as a silent infection until the adult worms lay eggs [35]. Liver is the most affected organ in schistosomiasis following oviposition when the eggs are trapped in the tissue. The trapped eggs within the hepatic tissue promote granuloma formation and cause fibrosis and vascular damage as well [36]. The trapped eggs in liver trigger an inflammatory cascade, causing liver damage associated with the increase of transaminases (AST and ALT) levels in serum [37]. This was mainly assigned to the irritation of hepatocytes by the released toxins or metabolic products of the growing worm, adult worms, and eggs [38]. Here, our results revealed that administration of both yogurt and probiotics were capable of decreasing transaminases levels in serum, which gives slight indication that both yogurt and probiotics protect the liver from schistosomiasis-induced injury. These results were in consistence with that of Kirpich and colleagues in which administration of probiotics results in the improvement of liver enzyme function in human alcohol-induced hepatic damage [39], and Ghanem et al. [20], who reported that yogurt supplementation recovered the activity of liver function parameters in infected mice to levels observed in control animals. Moreover, Mohamed et al. indicated that the combined treatment of PZQ and *L. sporogenes* decreases the hepatic and intestinal damage caused by *S. mansoni* infection [40].

It has been reported that probiotics has the ability to stimulate nonspecific host immune resistance to microbial pathogens results in dimension of worm burden [41, 42], which in turn decreases the number of eggs laid in hepatocytes and protecting liver from damage. In this context, the oral introduction of *L. casei* and *L. bulgaricus* stimulates macrophages and activates phagocytosis in mice [43]. Phagocytosis is an early inflammatory immune response mediated by phagocytes, which release some toxic agents including ROS and some lytic enzymes before the process of antibody production. Induction of liver stress due to *S. mansoni* infection is characterized by oxidant/antioxidant imbalance and elevated level of ROS. Wilson et al. [44] and de Oliveira et al. [45] reported that oxidative stress in liver tissue may arise as a result of several causes such as trapped eggs, changes in vascular tone, and soluble immune mediators. In addition, the stimulated leukocytes, eosinophils, macrophages, Kupffer cells, and the developing granulomas were considered as the major factors responsible for elevated oxidative stress in the hepatic tissue [45]. Mantawy et al. [46] concluded that a marked increase in immunoglobulins in the host results in the inability of the tissue to scavenge excessive ROS by the internal antioxidants, leading to oxidative stress and oxidation of various macromolecules such as lipids, proteins, and DNA. In accordance with this finding, we report an elevation in oxidative stress markers indicating an excessive generation of oxidants. We noticed also that the enzymatic antioxidant defense system failed to counteract excessive oxidant production. Increased levels of oxidative stress markers may stimulate hepatic stellate cells (HSCs) and collagen gene transcription, leading to liver fibrosis [47, 48]. Therefore, the discovery as well as the development of effective molecules able to modulate the disturbance in the antioxidant system associated with liver damage-induced by *Schistosoma* infection is highly recommended.

In the present study, we indicated a significant depletion in the antioxidant enzyme activities of SOD, CAT, GST, GPx, and GR in the hepatic tissue of mice infected with *S. mansoni*. However, probiotics or yogurt treatment prevented this inhibition. The antioxidant activity of different bacterial strains has been documented [49, 50]. Probiotic dahi, fermented milk containing *L. acidophilus* and *L*. *casei,* reduces oxidative stress in animal models [51]. This was mainly assigned to the suppressive effect of the tested probiotics on the NF-κB pathway [52], and the attenuated levels of circulating endotoxin [53]. Martarelli et al. [54] attributed the antioxidant activity of different probiotics to the ability of some bacterial stains to produce B vitamins. Among these vitamins B1, B5, and B6 are known antioxidants [55–57]. It has been found that administration of probiotics (300 g/day) containing *L. acidophilus* La5 and *B. lactis* Bb12 or traditional yogurt (300 g/day) for 6 weeks results in significant increase in the activity of both erythrocyte SOD and GPx in type 2 diabetes mellitus (T2DM) patients [58]. This finding was accompanied with an obvious increase in the total antioxidant status compared to the control group and decreased the serum MDA levels comparable to the baseline values.

Furthermore, probiotics exhibit an antioxidant property by scavenging ROS and activation of both SOD and CAT. These enzymes play a major role in the dimension of the deleterious effect of superoxide radicals and detoxification of H_2_O_2_ thus preventing cellular oxidative damage-induced by oxidative stress [59]. Additionally, it inhibits the infiltration and migration of neutrophil, which serve as the greatest source of ROS [60]. Probiotics can also block apoptosis and necrosis in hepatocytes by modifying Bax/Bcl-2 ratio, suppressing caspases activity, and reducing DNA damage [61, 62]. An in vivo study revealed that yogurt administration produced an anti-inflammatory effect and downregulated the expression of various inflammatory cytokines [63, 64].

We showed that the level of NO production was significantly increased in case of granuloma linked to *Schistosoma* infection, which is catalyzed by inducible NO synthase (iNOS) found in macrophages. The transcription factor represented in nuclear factor-kappa B (NF-κB) was found to control the expression of iNOS and increases in response to endotoxin, proinflammatory cytokines, and other factors [65]. Parola and Robino [66] stated that NO generated in tissue stimulates tissue fibrosis via activating fibrogenic cytokines which in turn increase collagen synthesis. However, yogurt and probiotics were able to prevent NO elevation in this study that is in accordance with the findings of Ulisse et al. [67] in which the nuclear localization of NF-κB was efficiently inhibited by administration of *Lactobacillus* species. The molecular mechanism that triggers NF-κB suppression was directly correlated to the activation of nuclear factor of kappa light polypeptide gene enhancer in B-cells inhibitor, alpha (Iκ-Bα), which has the ability to mask the nuclear localization signal (NLS) of NF-κB.

In the present study, the expression level of the apoptotic markers such as Bax mRNA and caspase-3 protein was increased in hepatic tissues of *S. mansoni* infected mice, whereas the expression level of *Bcl-2* mRNA was decreased. Moreover, activation of caspase-9 shifted the balance toward the expression of pro-apoptotic Bcl-2 family members, that subsequently activates caspase-3 and hence induction of apoptotic cell death [68]. The present findings agree with the findings of Duan et al. [69] that some factors from Schistosoma worms could reverse or minimize liver fibrosis by inducing cell apoptosis. An in vitro study in 2014 demonstrated that SEA inhibits the activation of HSC, which leads to the induction of controlled cell death program [70]. However, probiotic or yogurt treatment reduced the pro-apoptotic markers and elevated the level of anti-apoptotic markers. Our results agree with the results of Neish et al. [71] that probiotics have anti-apoptotic activity, which is assigned, to their ability to inhibit cytokine-induced apoptosis. Yan and Polk [72] showed that *L. rhamnosus* promotes the survival of intestinal epithelium through activating the anti-apoptotic Akt/protein kinase B and inhibiting the pro-apoptotic p38 MAP kinase. Lin et al. [73] revealed the anti-apoptotic effect of probiotics-fermented purple sweet potato yogurt by enhancing the survival of cardiac cells in hypertensive hearts. Moreover, a combined treatment of PZQ and *L. sporogenes* impairs chromosomal aberrations and DNA damage upon *Schistosoma* infection rather than treatment with PZQ alone [74].

Our results showed that the level of MMP-9 was markedly upregulated due to infection with *S. mansoni*. These results are in consistence with that of Chuah et al. [75] who confirmed the elevated level of MMP-9 in hepatic granuloma associated with *Schistosoma* infection. It was shown that MMPS play a central role in the progression of liver inflammatory processes. Both macrophages and T cell lymphocytes are considered as the major producer of MMPs including MMP-9 in which their expressions are modulated by several cytokines [76]. The main function of MMP-9 is the hydrolysis of the basement membrane, which is quit important for migration of neutrophils, eosinophils, and T cell lymphocytes as well as the release of different inflammatory cytokines [77]. Our results indicated that, probiotic or yogurt treatment markedly reduced MMP-9 protein expression in liver tissue (Additional file 1: Table S1) in accordance with the decreased MMP-9 activity in colon cancer cells following administration of *L. delbrueckii* as reported by Wan et al. [78]. Consumption of a multi-strain probiotic with a high fat (predominantly corn oil) diet was also found to elevate MMP-9 and minimizes systemic oxidative stress [76].

## Conclusions

In the current study, we investigated the protective effect of probiotics and yogurt on liver fibrosis and oxidative stress in mice infected with *S. mansoni*. Treatment with both yogurt and probiotics revealed significant anti-apoptotic, antioxidant, and decreased the granulaoma formation in hepatic tissue of *S. mansoni* infected mice. Despite the promising anti-schistosomal activities of probiotics and yogurt, further studies should be conducted to confirm this effect.

## Additional file

Additional file 1: **Table S1.** Effects of probiotics or yogurt administration on the number of MMP-9, and caspase-3 positive cells in hepatocytes of *S. mansoni*-infected mice. (DOC 33 kb)

## Abbreviations

ALT: Alanine aminotransferase
AST: Aspartate aminotransferase
CAT: Catalase
ECM: Extracellular matrix
GPx: Glutathione peroxidase
GR: Glutathione reductase
GSH: Glutathione
iNOS: Inducible NO synthase
LPO: Lipid peroxidation
MDA: Malondialdehyde
MMP: Matrix metalloproteinase
MMP9: Matrix metallopeptidase 9
NO: Nitric oxide
PZQ: Praziquantel
RNS: Reactive nitrogen species
ROS: Reactive oxygen species
SOD: Superoxide dismutase
