# Supplementary material for: Probiotics and yogurt modulate oxidative stress and fibrosis in livers of Schistosoma mansoni-infected mice
Source: BMC Complement Altern Med. 2019 Jan 3;19:3. doi: 10.1186/s12906-018-2406-3 (PMC6318950; doi:10.1186/s12906-018-2406-3)
Supplement: Supplementary file 1 — Table S1. Effects of probiotics or yogurt administration on the number of MMP-9, and caspase-3 positive cells in hepatocytes of S. mansoni-infected mice. (DOC 33 kb) [file 12906_2018_2406_MOESM1_ESM.doc]

**Table S1:** Effects of probiotics or yogurt administration on the number of MMP-9, and caspase-3 positive cells in hepatocytes of *S*. *mansoni*-infected mice.

| **Groups** | **Number of MMP-9 positive cells** | **Number of caspase-3 positive cells** |
| --- | --- | --- |
| **Control** | 3.4 ± 0.8 | 10.9 ± 3.1 |
| **Vehicle control** | 73.6 ± 4.3^#^ | 69.3 ± 3.9^#$^ |
| **PZQ** | 63.1 ± 5.8^#$^ | 61.8 ± 4.5^#$^ |
| **Pre-****Probiotics+infection** | 23.4 ± 2.1^#$^ | 12.5 ± 1.7^$^ |
| **Probiotics+infection** | 32.3 ± 4.6^#$^ | 22.6 ± 1.8^#$^ |
| **Pre-Yogurt+infection** | 11.2 ± 2.3^$^ | 9.8 ± 1.1^$^ |
| **Yogurt+infection** | 21.1 ± 3.4^#$^ | 23.4 ± 3.8^#$^ |

Values are means of total positive numbers from 10 random and continuous fields from each section ± SEM. ^#^p<0.05, significant change with respect to **Control** group; ^$^p<0.05, significant change with respect to **Vehicle control** group. **MMP-9**: matrix metallopeptidase 9.
